# 

**Published:** 1872-02

**Authors:** 


### CONTENTS.

COMMUNICATIONS.

Dental Jottings ........ 53
Therapeutical Action of Arsenic ........ 61

PROCEEDINGS OF SOCIETIES.

Discussion before the Sixth Annual Meeting of the Ohio State Dental Society ........ 65

EDITORIAL.

Old Colony Dental Society ........ 88
A Visit—Educational ........ 88
Still at School ........ 91
"Monsieur Tonsin Come Again"—Rubber Subject ........ 92
Celluloid Base ........ 94
Ohio Dental College Association ........ 94
Mississippi Valley Dental Society ........ 95

